# MiR-107 and miR-99a-3p predict chemotherapy response in patients with advanced colorectal cancer

**DOI:** 10.1186/1471-2407-14-656

**Published:** 2014-09-07

**Authors:** Sonia Molina-Pinelo, Amancio Carnero, Fernando Rivera, Purificacion Estevez-Garcia, Juan Manuel Bozada, Maria Luisa Limon, Marta Benavent, Javier Gomez, Maria Dolores Pastor, Manuel Chaves, Rocio Suarez, Luis Paz-Ares, Fernando de la Portilla, Andres Carranza-Carranza, Isabel Sevilla, Luis Vicioso, Rocio Garcia-Carbonero

**Affiliations:** Instituto de Biomedicina de Sevilla (IBiS), Hospital Universitario Virgen del Rocio/CSIC/Universidad de Sevilla, Manuel Siurot s/n, 41013 Seville, Spain; Department of Medical Oncology, Hospital Marqués de Valdecilla, Avda. Valdecilla s/n, Santander, Spain; Department of Gastroenterology, Hospital Universitario Virgen del Rocio, Avda. Manuel Siurot s/n, Sevilla, Spain; Department of Medical Oncology, Hospital Universitario Virgen del Rocio, Avda. Manuel Siurot s/n, Sevilla, Spain; Department of Pathology, Hospital Marqués de Valdecilla, Avda. Valdecilla s/n, Santander, Spain; Department of Surgery, Hospital Universitario Virgen del Rocio, Avda. Manuel Siurot s/n, Sevilla, Spain; Department of Medical Oncology, Hospital Virgen de la Victoria, Lugar Arroyo Teatinos s/n, Malaga, Spain; Department of Pathology, Hospital Virgen de la Victoria, Lugar Arroyo Teatinos s/n, Malaga, Spain

**Keywords:** MicroRNAs, Advanced colorectal cancer, Chemotherapy response, Prediction

## Abstract

**Background:**

MicroRNAs (miRNAs) are involved in numerous biological and pathological processes including colorectal cancer (CRC). The aim of our study was to evaluate the ability of miRNA expression patterns to predict chemotherapy response in a cohort of 78 patients with metastatic CRC (mCRC).

**Methods:**

We examined expression levels of 667 miRNAs in the training cohort and evaluated their potential association with relevant clinical endpoints. We identified a miRNA profile that was analysed by RT-qPCR in an independent cohort. For a set of selected miRNAs, bioinformatic target predictions and pathway analysis were also performed.

**Results:**

Eight miRNAs (let-7 g*, miR-107, miR-299-5p, miR-337-5p, miR-370, miR-505*, miR-889 and miR-99a-3p) were significant predictors of response to chemotherapy in the training cohort. In addition, overexpression of miR-107, miR-337-5p and miR-99a-3p, and underexpression of miR-889, were also significantly associated with improved progression-free and/or overall survival. MicroRNA-107 and miR-99a-3p were further validated in an independent cohort as predictive markers for chemotherapy response. In addition, an inverse correlation was confirmed in our study population between miR-107 levels and mRNA expression of several potential target genes (CCND1, DICER1, DROSHA and NFKB1).

**Conclusions:**

MiR-107 and miR-99a-3p were validated as predictors of response to standard fluoropyrimidine-based chemotherapy in patients with mCRC.

**Electronic supplementary material:**

The online version of this article (doi:10.1186/1471-2407-14-656) contains supplementary material, which is available to authorized users.

## Background

Colorectal cancer (CRC) is one of the most common malignant tumors worldwide [1]. Despite advances in early detection, about one third of patients present metastatic disease at diagnosis, and ~40% of those with early-stage tumors eventually relapse at some point over the course of the disease [2]. Systemic therapy is the mainstay of care for patients with metastatic CRC (mCRC) [3]. Several combination regimens including fluoropyrimidines and oxaliplatin and/or irinotecan, with or without monoclonal antibodies targeting VEGF or EGFR, have been successfully developed and are associated with response rates of 40-60% and a median survival of 20–24 months [4–9]. Despite the undeniable progress achieved, still a considerable proportion of patients do not respond to therapy and reliable tools to prospectively identify which patients are more likely to benefit are needed.

Several driver mutations have been identified to be relevant in CRC carcinogenesis [10, 11]. The most commonly involved pathways include the Wnt/β-catenin, TGF-β/BMP, TP53, receptor tyrosine kinase, KRAS and PI3K signaling pathways [10]. Many of these proteins are altered and seem to be affected by microRNA regulation. In this sense, the miR-135 family may play an important role in early CRC development as it down-regulates APC, leading to activation of the Wnt/β-catenin pathway [12]. On the other hand, the lethal-7 (let-7) family of miRNAs has been found to display tumor suppressor functions by repressing translation of KRAS. Interestingly, patients with KRAS-mutated CRC and high let-7 levels seem to benefit from EGFR-targeted agents, suggesting that let-7 expression could potentially counteract resistance mediated by RAS activating mutations [13]. KRAS has been also described to be a direct target of other miRNAs such as miR-143, miR-146b-3p, miR-18a, and miR-486-5p [14–17] and miR-126 has been implicated in PI3K signalling [18]. Other miRNAs known to be involved in CRC pathogenesis affect epithelial differentiation (miR-141 and miR-200c), WNT signaling (miR-145, miR-135a and miR-135b), and migration and invasion (miR-21, miR-373 and miR-520c) [19–22].

From a clinical perspective, several studies have identified groups of miRNAs with potential utility for early diagnosis or prognostic stratification of CRC patients. However, there are no robust studies to evaluate the potential ability of miRNA to predict response to selected chemotherapy regimens. Based on these premises, the purpose of this study was to evaluate the ability of miRNA expression patterns to predict chemotherapy response in patients with mCRC treated with fluoropyrimidine-based standard chemotherapy regimens.

## Methods

### Patients and tumor samples

Patients that met the following inclusion criteria were selected for the present study: (1) histologically confirmed diagnosis of primary CRC; (2) TNM stage IV; (3) fluoropyrimidine-based first-line chemotherapy for advanced disease; (4) measurable disease per RECIST criteria; (5) adequate clinical data recorded in medical charts; (6) adequate tissue specimen available (snap-frozen at -80°C with a proportion of tumor cells > 50%). This study was approved by the ethics committees of Hospital Universitario Virgen del Rocio (Sevilla), Hospital Marques de Valdecilla (Santander) and Hospital Virgen de la Victoria (Malaga), and all patients provided written informed consent prior to study entry.

Tumor tissue samples of 78 patients were collected at the Hospital Universitario Virgen del Rocio (Sevilla), Hospital Marques de Valdecilla (Santander), Hospital Virgen de la Victoria (Malaga) and Hospital de la Merced (Osuna). Main characteristics of study population are summarized in Table 1 and are representative of a standard metastatic CRC population. The majority of patients (96%) were treated with a chemotherapy regimen that included fluoropyrimidines and either oxaliplatin (76%) or irinotecan (20%). The patient population was divided in a training cohort (N = 39) that was used for miRNA profile development and an independent validation cohort (N = 39).

**Table 1:** **Characteristics of study population**

	Training cohort (N = 39)	Validation cohort (N = 39)
**Age, years – median [range]**	62 [54–70]	66 [61–72]
**Gender - N(%)**		
Male	23 (59.0%)	29 (74.4%)
Female	16 (41.0%)	10 (25.6%)
**Histology of primary tumor - N(%)**		
Adenocarcinoma	35 (89.7%)	39 (100%)
Mucinous adenocarcinoma	4 (10.3%)	-
**Chemotherapy regimen - N(%)**		
Ox/FP regimens	30 (76.9%)	29 (74.3%)
Ir/FP regimens	7 (17.9%)	9 (23.1%)
FP monotherapy	2 (5.2%)	1 (2.6%)
**Response to chemotherapy - N(%)**		
Objective Response (CR, PR)	18 (46.2%)	24 (61.5%)
No Response (SD, PD)	21 (53.8%)	15 (38.5%)
**Survival, months – median [range]**		
Progression-free survival	12.2 [6.3-18.9]	11.6 [8.6-18.3]
Overall survival	24.6 [15.8-37.2]	21.5 [13.3-31.1]

Continuous variables are expressed as median [interquartile range (IQR)] and categorical variables as number of cases (%). Ox: oxaliplatin; FP: fluoropyrimidine; Ir: Irinotecan. CR: complete response; PR: partial response; SD: stable disease; PD: progressive disease.

### Clinical outcome variables and statistical analysis

Descriptive statistics were used to characterize the most relevant clinical parameters. The association of categorical variables was explored by the chi-squared test or Fisher’s exact test. To assess distribution of continuous variables among study groups parametric (t-test) or non-parametric tests (Kruskal-Wallis or Mann–Whitney tests) were employed when appropriate.

Tumor response was evaluated by conventional methods according to the standard RECIST 1.0 criteria: a complete response (CR) was defined as the disappearance of all measurable and evaluable evidence of disease; a partial response (PR) was defined as a ≥ 30% decrease in the sum of the longest diameters of target lesions; stable disease (SD) was considered if the tumor burden decreased less than 30% or increased less than 20%; and progressive disease (PD) was indicated by a >20% increase in the sum of the longest diameters of target lesions or the appearance of any new lesion. Patients were classified according to best response to chemotherapy in two groups: those that achieved an objective response (Responders [R]: CR + PR) and those that did not (Non-responders [NR]: SD + PD). Progression Free Survival (PFS) was defined as the time elapsed from the date of initiation of first-line chemotherapy to the date of the first documented evidence of disease progression. Overall survival (OS) was calculated from the start of therapy for advanced disease to the date of death from any cause. The Kaplan-Meier product limit method was used to estimate time-dependent variables (PFS and OS), and differences observed among patient subgroups were assessed by the log rank test. Multivariate analyses were performed using the Cox proportional hazards model. P < 0.05 was considered significant. All analyses were performed using the Statistical Package for the Social Sciences software (SPSS 17.0 for Windows; SPSS Inc, Chicago, IL).

### RNA isolation and miRNA qRT-PCR assay

Total RNA, containing small RNA, was extracted from tumor tissue samples by mirVana miRNA isolation kit (Ambion, Austin, TX, USA) according to the manufacturer’s instructions. Mature human miRNA expression was detected and quantified using the TaqMan® Low Density Arrays (TLDA) based on Applied Biosystems’ 7900 HT Micro Fluidic Cards (Applied Biosystems, CA, USA) following instructions provided by the manufacturer. The Human MicroRNA Card Set v2.0 array is a two card set containing a total of 384 TaqMan® MicroRNA Assays per card to enable accurate quantification of 667 human microRNAs, all catalogued in the miRBase database. TLDAs were performed in a two-step process, as previously described [23].

Eight miRNAs (let-7 g*, miR-107, miR-299-5p, miR-337-5p, miR-370, miR-505*, miR-889 and miR-99a-3p), which were selected because their expression in the Taqman Low Density Array card assays was significantly associated with response to chemotherapy and clinical outcome, were further analyzed in an independent validation cohort by qPCR. For this, RNA was reverse transcribed to cDNA using TaqMan® MicroRNA Assays (Applied Biosystems, CA, USA). Ten ng of total RNA were reverse transcribed using the TaqMan miRNA reverse transcription kit in a total volume of 15 μl, according to the manufacturer's protocol. The reactions were incubated for 30 min at 16°C, 30 min at 42°C, and 5 min at 85°C, and then kept at 4°C. Thereafter, 1.33 μL of cDNA was used for TaqMan MicroRNA Assays. The reactions were incubated at 95°C for 10 min, followed by 40 cycles of 15 sec at 95°C and 1 min at 60°C. All experiments were performed in triplicate.

### Analysis of miRNA expression profiles

Expression of target miRNAs was normalized to the expression of MammU6, the most widely-used endogenous miRNA control for RT-qPCR in the literature. One non-human miRNA was used in each experiment as a negative control. Finally, the cards were processed and analyzed on an ABIPrism 7900 HT Sequence Detection System. Cycle threshold (Ct) values were calculated with the SDS software v.2.3 using automatic baseline settings and a threshold of 0.2. Relative quantification of miRNA expression was calculated by the 2^-ΔΔCt^ method (Applied Biosystems user bulletin no.2 (P/N 4303859)). MicroRNAs expression was computed using Real-Time Statminer© software v.4.2 (Integromics, Inc). This software performs a moderate t-test between the groups (R versus NR) and corrects them using the Benjamini-Hochberg algorithm with the False Discovery Rate (FDR) set at a value of 5%. For undetected miRNAs with Ct values beyond the maximum Ct 36, the StatMiner software imputed a value set to the maximum Ct. For the purpose of this study, significant miRNA expression was considered only when miRNAs were detected in at least 50% of samples in each group being compared. The raw and normalized TaqMan array data have been deposited in the Gene Expression Omnibus under the accession number GSE48664.

Experimentally verified mRNA by previous research were determined using the web-accessible information resource miRWalk [24]. We then validated 9 potential target genes according to expression levels of mir-107 by Taqman real-time RT-PCR assay (Applied Biosystems, CA, USA). Expression of miR-107 was normalized to the expression of MammU6. Pearson's correlation coefficient was used to assess the linear association of miRNA and target mRNA expression (SPSS 17.0 for Windows; SPSS Inc, Chicago, IL).

### 3′-UTR reporter assay for miR target validation

Confirmation of miR-107-binding to the 3′-UTR of CCDN1. HEK 293 cells at 80% confluency were co-transfected with luciferase reporter plasmids harboring the complete 3′-UTR of the desired gene (SwitchGear Genomics) along with 100nM of miR107-mimic or miRNA control (Sigma). DharmaFECT Duo (Thermo Scientific) was used as the transfection reagent in Opti-MEM (Life Technologies). Luminescence was assayed 24 hours later using LightSwitch Assay Reagents (SwitchGear Genomics) according to the manufacturer's instructions. Knockdown was assessed by calculating luciferase signal ratios for specific miRNA/non-targeting control, using empty reporter vector as control for non-specific effects. Each experiment was performed in triplicate

## Results

### MicroRNA profile development

#### MicroRNA expression patterns according to objective response to chemotherapy

The relative miRNA expression levels for patients that achieved an objective response to chemotherapy (R) versus those that did not (NR) are represented in Additional file 1: Figure S1. Of the 667 miRNAs assessed, 7% (N = 46) were differentially expressed (p < 0.05) among these two subgroups described (R versus NR). However, only eight of these 46 miRNAs were detected in at least 50% of tested samples (let-7 g*, miR-107, miR-299-5p, miR-337-5p, miR-370, miR-505*, miR-889 and miR-99a-3p) (Table 2), and were therefore considered to be representative of the general behaviour of the study population.

**Table 2:** **Differently expressed miRNAs by objective response to chemotherapy (Training Cohort)**

MicroRNAs	R vs NR (-ΔΔCt)	Adjusted p-values*
let-7 g*	0.863	**0.042**
miR-107	0.706	**0.042**
miR-299-5p	0.864	**0.006**
miR-337-5p	0.952	**0.018**
miR-370	1.162	**< 0.001**
miR-505*	0.877	**0.006**
miR-889	-0.560	**0.042**
miR-99a-3p	0.715	**0.016**

R – responders to chemotherapy (complete or partial response); NR – non-responders to chemotherapy (stable or progressive disease) (RECIST criteria).

*p values adjusted for multiple testing by Benjamini-Hochberg method. The bold value indicates a statistically significant result.

#### Impact of selected miRNAs expression on progression free and overall survival

**Figure 1:**
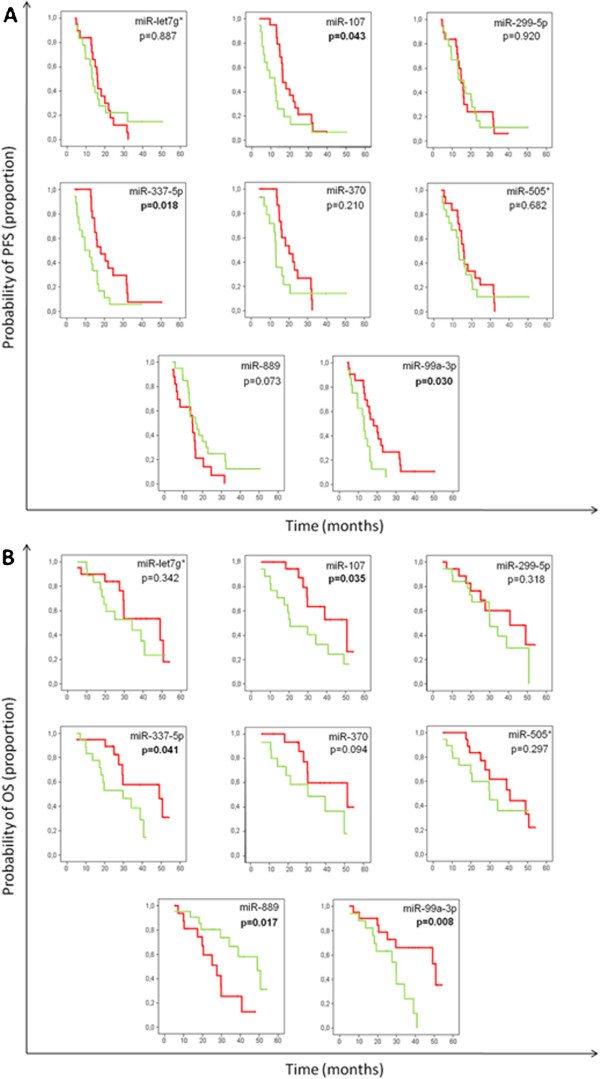
**Training cohort: Clinical outcome of patients by miRNA expression levels. (A)** Progression-free survival (PFS) and **(B)** Overall survival. The solid red line represents patients with higher miRNA expression levels (above the median). The solid green line represents patients with lower miRNA expression levels.

These selected miRNAs able to predict response to chemotherapy were further assessed to evaluate their potential association with progression free survival (PFS) and overall survival (OS) of patients. Overall, median PFS was 13.6 months [range: 8.8-21.2] and median OS was 25.6 months [range: 17.1-39.3], consistent with survival data reported in the literature for this patient population. Kaplan-Meier estimates for PFS and OS according to miRNA expression levels grouped as above or below the median are shown in Figure 1A and B, respectively. Among tested miRNAs, expression of miR-107, miR-337-5p and miR-99a-3p was significantly associated with both PFS and OS (p < 0.05), while that of miR-889 was only associated with OS (p < 0.05). In addition, a trend of borderline significance was observed for miR-370 with OS (p = 0.094).

Multivariate analyses confirmed miR-107, miR-337-5p and miR-99a-3p as independent predictive factors for PFS. Regarding overall survival, only miR-889, together with age and sex retained independent prognostic significance in the Cox multiple regression model (Table 3).

**Table 3:** **Univariate and multivariate analysis of predictive miRNA for PFS and OS in metastatic colorectal cancer patients (Training Cohort)**

VARIABLES	PFS				OS			
	Univariate Analysis		Multivariate Analysis		Univariate Analysis		Mutivariate Analysis	
	HR (95% CI)	***p-value***	HR (95% CI)	***p-value***	HR (95% CI)	***p-value***	HR (95% CI)	***p-value***
Age	0.99 [0.95-1.02]	0.357	0.99 [0.95-1.04]	0.765	1.01 [0.96-1.05]	0.746	1.06 [1.01-1.12]	**0.027**
Sex	0.51 [0.24-1.07]	0.069	0.60 [0.26-1.40]	0.232	0.42 [0.16-1.10]	0.069	0.17 [0.05-0.54]	**0.003**
miR-107	2.12 [1.05-4.29]	0.043	2.52 [1.18-5.42]	**0.017**	2.65 [1.06-6.67]	0.035	2.61 [0.86-7.92]	0.091
miR-337-5p	2.27 [1.12-4.58]	0.018	3.02 [1.34-6.83]	**0.008**	2.53 [0.95-6.80]	0.018	1.40 [0.42-4.69]	0.584
miR-99a-3p	2.34 [1.11-4.93]	0.030	2.50 [1.00-6.05]	**0.050**	3.46 [1.26-9.53]	0.008	1.99 [0.62-6.36]	0.243
miR-889	0.45 [0.22-0.94]	0.073	0.40 [0.16-0.90]	**0.027**	0.26 [0.10-0.71]	0.017	0.15 [0.04-0.47]	**0.001**

PFS: progression free survival; OS: overall survival; CI: confidence interval; HR: Hazard Ratio.

The bold value indicates a statistically significant result.

### Independent validation

**Figure 2:**
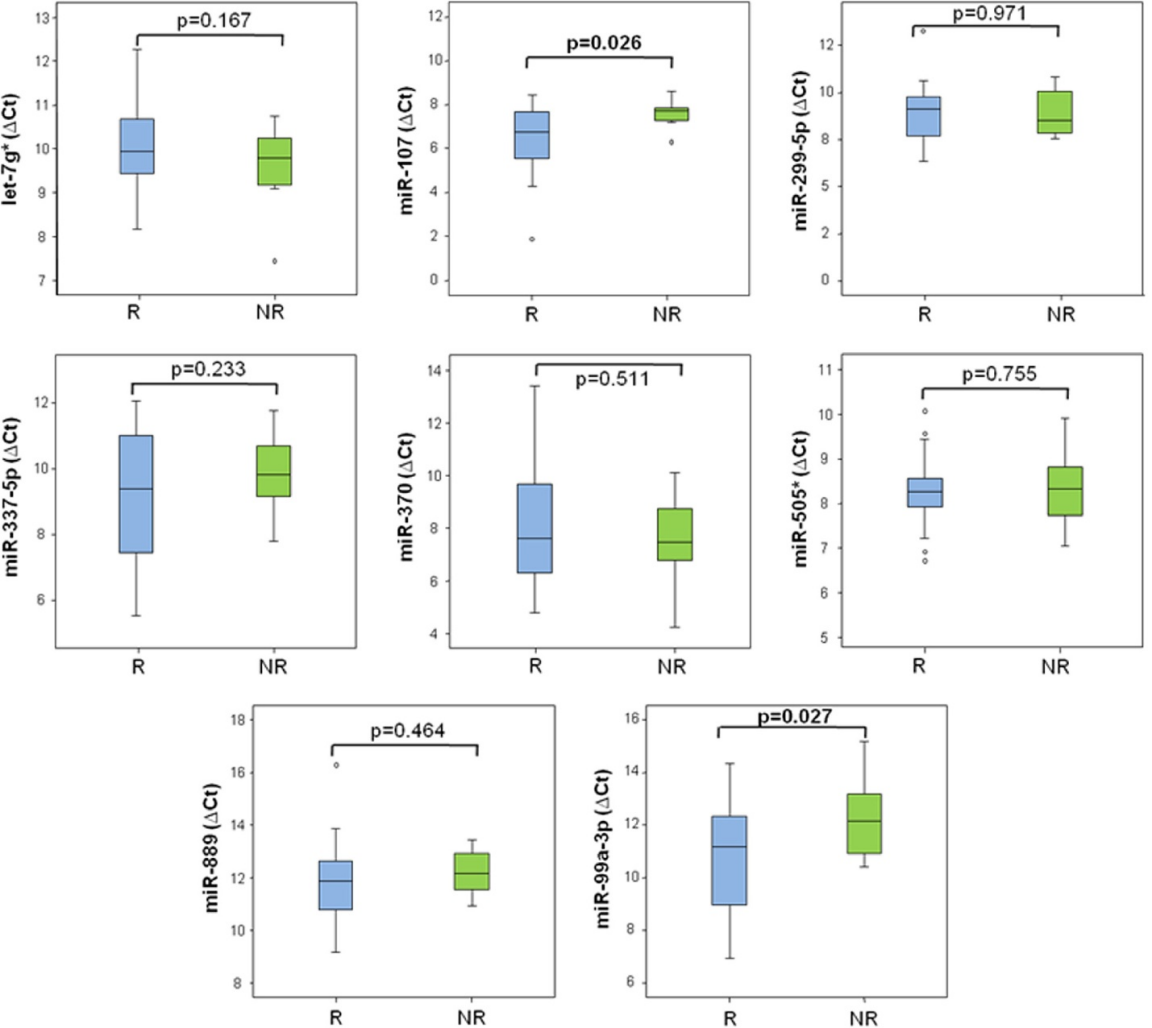
**Validation cohort: Median ΔCt values of validated miRNAs in patients with objective response to chemotherapy responders versus non-responders.** *p-value < 0.05. Data derived from RT-qPCR are presented as ΔCt values, with higher values standing for lower miRNA-expression. R: Responders; NR: Non-Responders.

As depicted in Figure 2, miRNA expression patterns in this validation cohort were consistent with those quantified in the training cohort, in the sense that similar association trends were observed between over- or under-expression of miRNAs and response to therapy. However, this association only achieved statistical significance for miR-107 and miR-99a-3p, with higher expression levels in mCRC patients that achieved an objective response to chemotherapy as compared to those that did not (p = 0.026 and p = 0.027, respectively).

### MicroRNA target prediction

**Figure 3:**
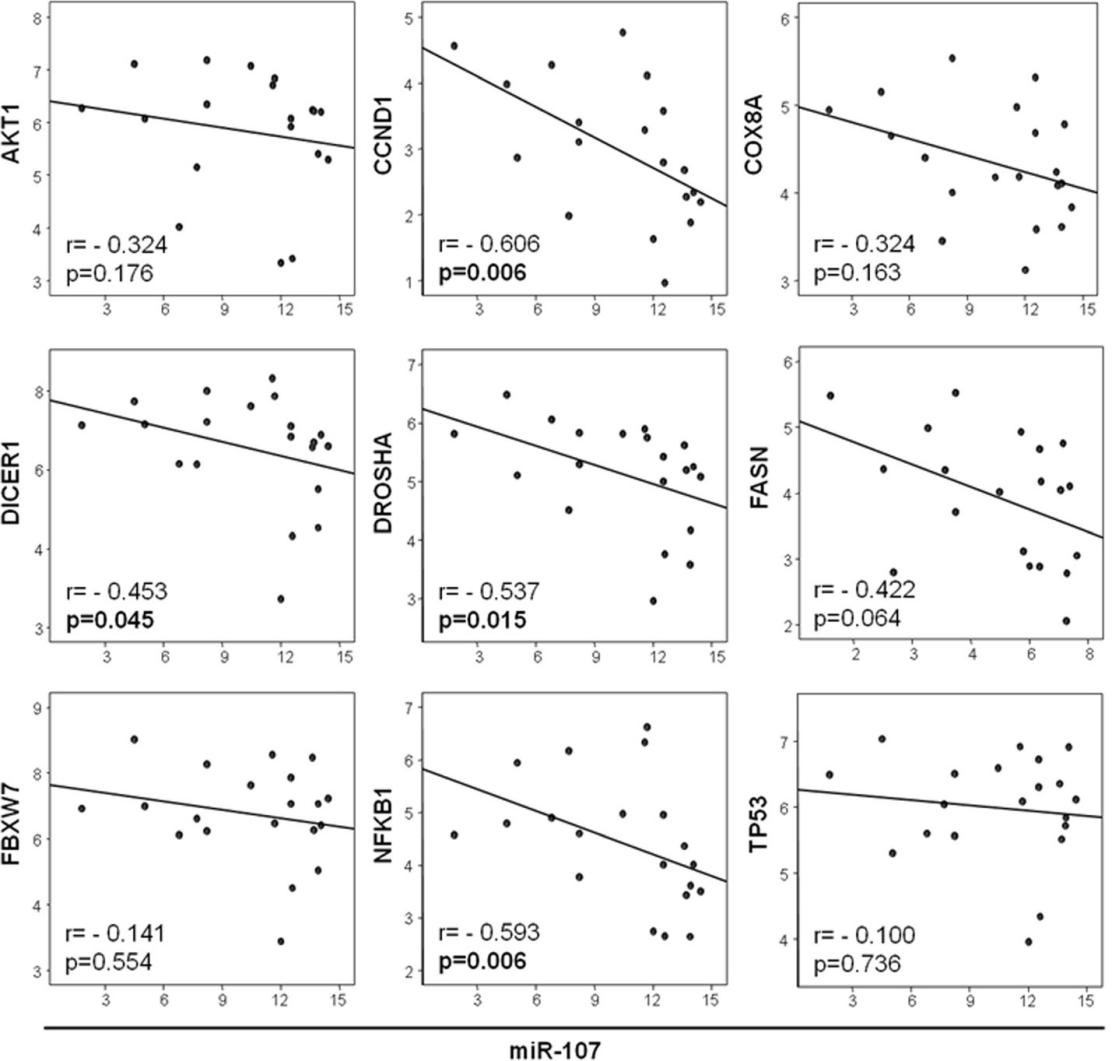
**Negative correlation between several potential target genes and miR-107 expression.**

**Figure 4:**
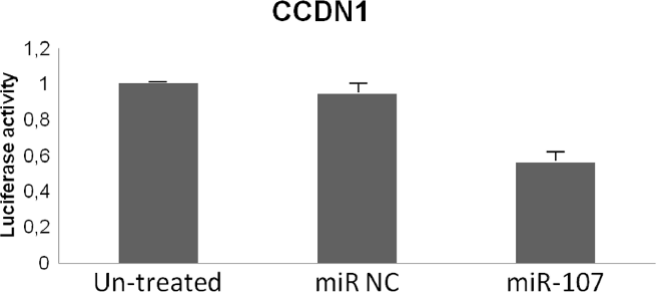
**3′-UTR reporter assay for miR target validation. HEK 293 cells were transfected with luciferase reporter vector containing the 3′-UTR region of CCDN1.** Reporter vectors were co-transfected with a miR-107 mimic or control miRNA mimic (miR NC). Following 24 h incubation, luciferase activity was measured.

A bioinformatic approach was used to identify experimentally verified target mRNAs of the validated miRNAs in our series, miR-107 and miR-99a-3p. However, whereas a number of genes have been experimentally validated to date for miR-107, none were identified for miR-99a-3p. Among the former, 9 of the miR-107 potential target genes were selected for further validation in our cohort, including genes involved in the PI3K/Akt signaling pathway and in the RNA-interference processing machinery. MicroRNA-107 target genes assessed were *AKT1 (*v-akt murine thymoma viral oncogene homolog 1)*, CCND1 (*cyclin D1)*, COX8A (*cytochrome c oxidase subunit VIIIA)*, DICER1 (*dicer 1, ribonuclease type III)*, DROSHA (*drosha, ribonuclease type III)*, FASN (*fatty acid synthase)*, FBXW7 (*F-box and WD repeat domain containing 7)*, NFKB1 (*nuclear factor of kappa light polypeptide gene enhancer in B-cells 1)*, and TP53 (*tumor protein p53). As depicted in Figure 3, an inverse correlation was observed between these nine mRNAs and miR-107 expression levels, being this correlation significant for *CCND1, DICER1, DROSHA and NFKB1*. Therefore, in individual tumor samples, higher levels of miR-107 were associated with lower levels of these targets. Subsequently, CCDN1 target was quantified using luciferase reporter gene assays. We observed that overexpression of miR-107 in HEK 293 cells significantly down-regulated the luciferase activity of reporter construct containing the CCDN1 3′-UTR (Figure 4). This data indicate that miR-107 binds directly to this target RNA and inhibits its expression, further supporting a potential role for miR-107 in the regulation of these genes.

## Discussion

In this study, we have evaluated global miRNA expression patterns in mCRC patients treated with fluoropyrimidine-based standard chemotherapy regimens. We identified eight miRNAs (let-7 g*, miR-107, miR-299-5p, miR-337-5p, miR-370, miR-505*, miR-889 and miR-99a-3p), the expression of which was significantly associated with response to chemotherapy. In addition, overexpression of miR-107, miR-337-5p and miR-99a-3p, and underexpression of miR-889, were also significantly associated with improved progression-free and/or overall survival. Moreover, miR-107 and miR-99a-3p were further validated in an independent cohort as predictive markers for chemotherapy response. This is to our knowledge the first study to assess the predictive role of miRNA expression profiles in patients with advanced CRC treated with fluoropyrimidines in combination with either oxaliplatin (77%) or irinotecan (18%), the most commonly used chemotherapy regimens in the treatment of this disease.

Altered miR-107 expression has been involved in several cancer types, including head and neck squamous cell carcinoma (HNSCC), ovarian, gastric or breast cancer, among others [25–27]. Our results have demonstrated that expression of this miRNA significantly influences sensitivity to fluoropyrimidine-based chemotherapy in patients with advanced colorectal cancer. miR-107 transcription is induced by p53 and it seems to function as a tumor suppressor gene in HNSCC cell lines through downregulation of protein kinase Cϵ (PKCϵ) [25]. PKCϵ is elevated in HNSCC and has been associated with a more aggressive phenotype [28]. Consistent with this, other groups have reported a tumor suppressor function for miR-107 in other cancer models including bladder, colon and pancreatic cancer. With regard to human colon cancer, miR-107 has been shown to regulate tumor angiogenesis by targeting hypoxia inducible factor-1β (HIF-1β) [29]. Indeed, overexpression of miR-107 in HCT116 colon cancer cells suppressed angiogenesis, tumor growth and tumor VEGF expression in mice. Decreased tumor angiogenesis induced by miR-107 may make tumor cells more vulnerable to a variety of cellular insults including genotoxic stress induced by DNA-damaging agents (i.e. conventional cytotoxic chemotherapy). In fact, antiangiogenic drugs such as the VEGF-targeting agents bevacizumab or aflibercept have demonstrated to be synergistic in combination with fluoropyrimidine-based chemotherapy in patients with advanced colorectal cancer. Moreover, other authors have shown that, compared with wild type tumors, tumors that lack HIF-1α are poorly vascularized but are faster growing, perhaps because of a loss of dependency upon neovascularization. These findings would be consistent with the increased response rate and improved prognosis observed in our series for patients over-expressing miR-107 [30, 31]. In addition, overexpression of miR-107 has been recently shown in gastric cancers in comparison with normal tissue, and up-regulation of these miRNA increased the proliferation of gastric cancer cells [32]. In colon cancer models some authors have reported that miR-103/107 may promote metastasis by targeting the metastasis suppressors DAPK and KLF4 [33]. They also found that, in the clinical setting, the signature of a miR-103/107 high, DPAK and KLF4 low expression profile correlated with the extent of lymph node and distant metastasis. However, no information was provided this study regarding relevant characteristics of the patient population such as stage of disease or therapeutic interventions. The discrepancies observed related to miR-103/107 function could be attributed to tissue- or context–specific effects, or may simply reflect the great complexity governing intra- and inter-cellular signaling networks. On the other hand, the precise role in cancer of the other validated miRNA in our series, miR-99a-3p, remain greatly unknown to date.

To explore the potential biological function of miR-107, we then identified validated targets using the computational prediction algorithm from miRWalk [24]. *AKT1, CCND1, DICER1, DROSHA, FASN, FBXW7, NFKB1 and TP53* are involved in several key pathways relevant to cancer such as the PI3K/Akt pathway and the miRNA-processing machinery [34–39]. As expected, we confirmed in individual tumor samples of our patients an inverse correlation of these target mRNA and miR-107 expression levels, being this correlation significant for *CCND1, DICER1, DROSHA and NFKB1*. These results may be considered a further validation of the functional role of miR-107 in the transcriptional regulation of these key genes in cancer.

## Conclusions

Our study has identified that miR-107 and miR-99a-3p may be used to predict response to therapy with standard fluoropyrimidine-based chemotherapy regimens in patients with mCRC. These results underline the great potential of miRNAs as novel biomarkers for personalized treatment strategies and also as potential therapeutic targets. Moreover, given the fact that CRC cells may release aberrantly expressed miRNAs into peripheral blood, miRNA profiling could also have a great potential as a minimally-invasive tool for prediction or monitoring of therapeutic outcome.

## Electronic supplementary material

Additional file 1: Figure S1: Volcano plot of differentially expressed miRNAs among responders versus non-responders to chemotherapy. The log_2_ of fold change is represented on the x-axis and the negative log of p-values from the t-test is represented on the y-axis. Dots above the dashed line have a p-value < 0.05 and points below that line have a *p*-value > 0.05. (TIFF 2 MB)
