# Supplementary figures and images for: MiR-107 and miR-99a-3p predict chemotherapy response in patients with advanced colorectal cancer
Source: BMC Cancer. 2014 Sep 7;14:656. doi: 10.1186/1471-2407-14-656 (PMC4167263; doi:10.1186/1471-2407-14-656)

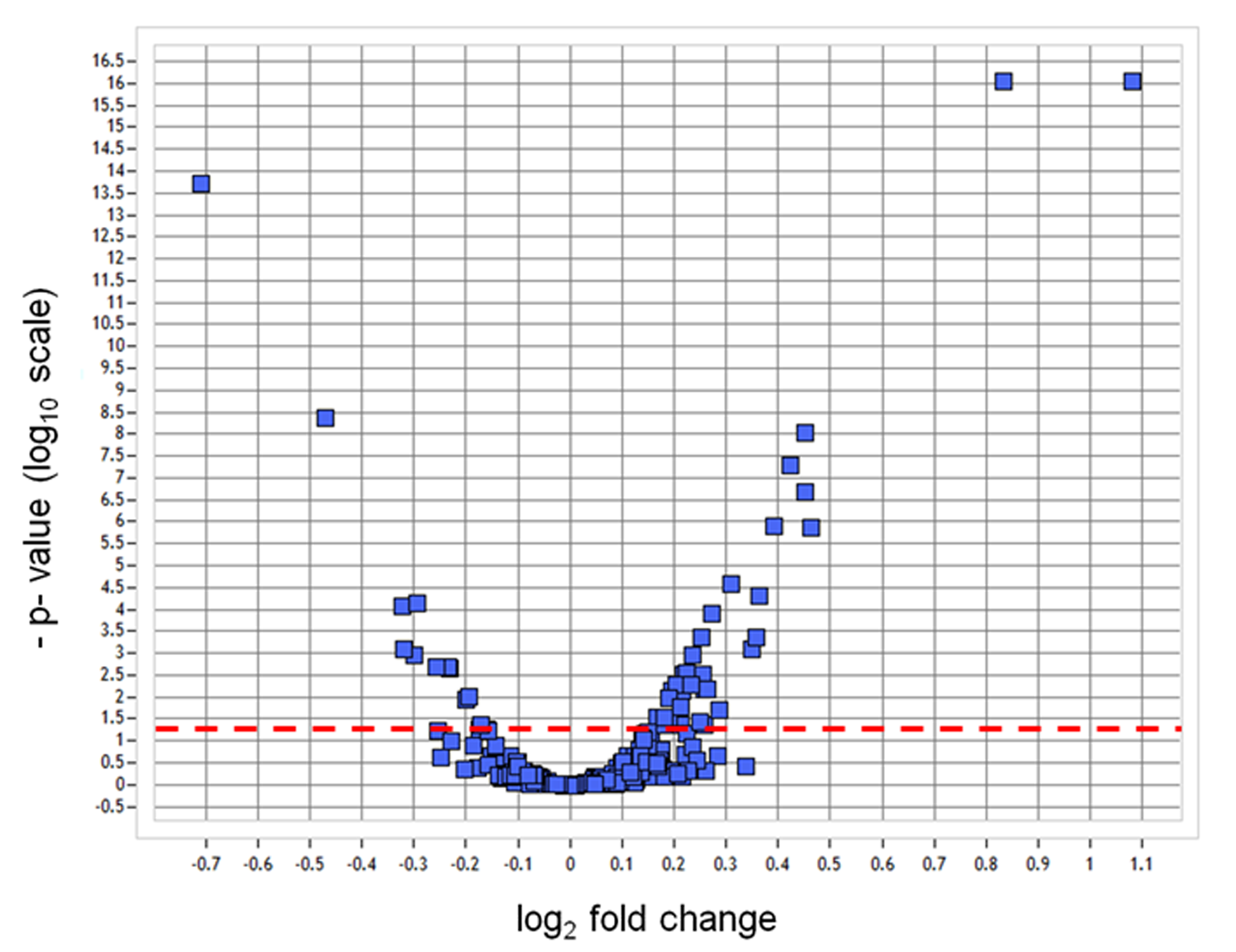

Supplement: Supplementary file 1 — Additional file 1: Figure S1: Volcano plot of differentially expressed miRNAs among responders versus non-responders to chemotherapy. The log2 of fold change is represented on the x-axis and the negative log of p-values from the t-test is represented on the y-axis. Dots above the dashed line have a p-value < 0.05 and points below that line have a p-value > 0.05. (TIFF 2 MB) [file 12885_2014_4838_MOESM1_ESM.tiff]
